# Detection of infectious disease outbreaks in twenty-two fragile states, 2000-2010: a systematic review

**DOI:** 10.1186/1752-1505-5-13

**Published:** 2011-08-23

**Authors:** Catherine Bruckner, Francesco Checchi

**Affiliations:** 1 Faculty of Infectious and Tropical Diseases, London School of Hygiene and Tropical Medicine, Keppel Street, London WC1E7HT, UK

## Abstract

Fragile states are home to a sixth of the world's population, and their populations are particularly vulnerable to infectious disease outbreaks. Timely surveillance and control are essential to minimise the impact of these outbreaks, but little evidence is published about the effectiveness of existing surveillance systems. We did a systematic review of the circumstances (mode) of detection of outbreaks occurring in 22 fragile states in the decade 2000-2010 (i.e. all states consistently meeting fragility criteria during the timeframe of the review), as well as time lags from onset to detection of these outbreaks, and from detection to further events in their timeline. The aim of this review was to enhance the evidence base for implementing infectious disease surveillance in these complex, resource-constrained settings, and to assess the relative importance of different routes whereby outbreak detection occurs.

We identified 61 reports concerning 38 outbreaks. Twenty of these were detected by existing surveillance systems, but 10 detections occurred following formal notifications by participating health facilities rather than data analysis. A further 15 outbreaks were detected by informal notifications, including rumours.

There were long delays from onset to detection (median 29 days) and from detection to further events (investigation, confirmation, declaration, control). Existing surveillance systems yielded the shortest detection delays when linked to reduced barriers to health care and frequent analysis and reporting of incidence data.

Epidemic surveillance and control appear to be insufficiently timely in fragile states, and need to be strengthened. Greater reliance on formal and informal notifications is warranted. Outbreak reports should be more standardised and enable monitoring of surveillance systems' effectiveness.

## Introduction

The World Bank describes a fragile state as a country 'facing particularly severe development challenges such as weak institutional capacity, poor governance, political instability, and frequently ongoing violence or the legacy effects of past severe conflict' [1].

In 2009, 29 countries were considered fragile, comprising a sixth of the world's population [2,3]. Fragile states generally feature poor health indicators, high malnutrition prevalence, scarcity of skilled health workers and worsening rates of extreme poverty [4-6]. Their populations are also highly vulnerable to infectious disease outbreaks, a reflection of inadequate government services and armed conflict-related phenomena such as forced displacement [7]. It has been suggested that most major epidemics worldwide occur in complex emergency and/or natural disaster settings [8].

Detection and early containment of outbreaks in these settings is also challenging, as highlighted by the Global Polio Eradication Initiative's recent setbacks in several fragile states, where genetic analysis has demonstrated previously undetected poliovirus transmission of one year or more duration [9]. Given the intensity of polio surveillance compared to other epidemic detection systems, it is plausible that many other disease outbreaks are detected late or not at all in these same settings.

The importance of epidemic surveillance is recognised, but there is a scarcity of evidence on optimal ways to detect outbreaks in the unique situations of fragile states, where routine health information systems are weak, diagnostic tools limited and resources for structured surveillance, such as training, sample transport and data transmission, very constrained. It has been suggested, at least for early warning systems in humanitarian emergencies, that emphasis should be placed on detecting alerts from health facilities or other informal sources (e.g. community informants and the media), rather than on analysis of weekly or other surveillance data, which often feature low completeness and timeliness, or high background noise due to non-specific case definitions [10]. So as to contribute to the evidence basis, we carried out a review of how outbreaks have been detected in 22 states that consistently met definitions of fragility over the past decade, and of the timeliness of alert and response processes.

## Methods

A systematic review of the published literature was performed to identify reports describing infectious disease outbreaks which began after 31^st ^December 1999, within a predefined list of fragile states. The list of fragile states was created using the World Bank's quantitative definition, taking into account both the eligibility of a country to receive an interest-free International Development Association loan and a nation's Country Policy and Institutional Assessment score [11]. Countries which met this definition for at least ten out of eleven years from the year 2000 to 2010 (see Additional File 1) were included in this study [2,12-15]. The final list of fragile states included in the review comprised Afghanistan, Angola, Burundi, the Central African Republic, Chad, Comoros, the Democratic Republic of the Congo, Guinea, Guinea-Bissau, Haiti, Liberia, Myanmar, the Republic of the Congo, Sao Tome et Principe, Sierra Leone, the Solomon Islands, Somalia, Sudan, Tajikistan, Timor-Leste, Togo and Zimbabwe.

Between 28^th ^July 2010 and 23^rd ^August 2010, a combined OVID SP search of the MEDLINE, EMBASE and Global Health databases was done. OVID SP is a search engine that taps into various literature databases relevant for global health. MEDLINE is a database of life sciences and biomedical journals. EMBASE is similar to MEDLINE but focuses on drug therapeutic studies. Global Health focuses on public health and medical science and includes conference abstracts, thesis reports, electronic information and other hard to find material. The basic search concepts were '(fragile state of interest) AND (epidemic-prone event) AND (detection)'. Each concept was expanded and variations of terms, including contemporary and historic, French and Spanish were included (see Additional File 1). Limitations applied were 'from 2000 to present' and 'humans'.

Outbreak descriptions were excluded from the search if they primarily involved foreign military forces, or if the disease of interest was HIV or poliomyelitis, due to the specific nature of surveillance for these two diseases.

Reports were included in the review if the circumstances of initial detection of the outbreak were reported; and/or if the time from onset to detection of the outbreak (determined using the definition in Table 1) could be calculated. Whenever this information was not clear based on the published report, we emailed the corresponding author once so as to solicit the missing information. We excluded the report if authors did not reply or could not provide the information requested.

**Table 1:** Definitions used for dates of interest in the outbreak timeline

Event	Definition
Onset	For diseases of which one case constitutes a potential outbreak, the date of onset of symptoms of the primary case. For diseases that are normally endemic but are considered epidemic when an unusual increase in burden is observed, the date on which the outbreak threshold was crossed, according to the authors. If investigation revealed previous undetected outbreaks of the same health event, this was also noted.

Detection	The date a report of a possible outbreak was sent to the highest appropriate level of authority. This could be the date of initial detection, if no authorities were required to be notified.

Confirmation	The date on which the aetiologic agent of the outbreak was confirmed.

Investigation	The date an investigation team arrived to the outbreak-affected community.

Declaration	The date the outbreak was officially declared as such by health authorities of the country concerned.

Control	The first day of a reactive vaccination campaign (we only computed the date of this event for diseases for which vaccination was the main control intervention available, since the date of implementation of other control interventions, such as water and sanitation, is difficult to define).

For each eligible outbreak, the mode of detection was categorised into (i) *data analysis *if an existing surveillance system detected the outbreak by noticing a temporal increase in aggregate incidence, either above a pre-determined threshold or at levels considered unusual compared to the baseline; (ii) *formal notification *if the initial alert was raised by health workers as part of an ongoing surveillance system; and (iii) *informal notification *if the alert was raised through mechanisms other than an existing surveillance system, either by health workers or other community members. Both authors made this classification independently and came to a consensus decision on any discrepant choices.

Whenever available, we also calculated time lags from detection or onset to further events in the outbreak timeline, as per the definitions in Table 1.

## Results

### Search strategy results

**Figure 1:**
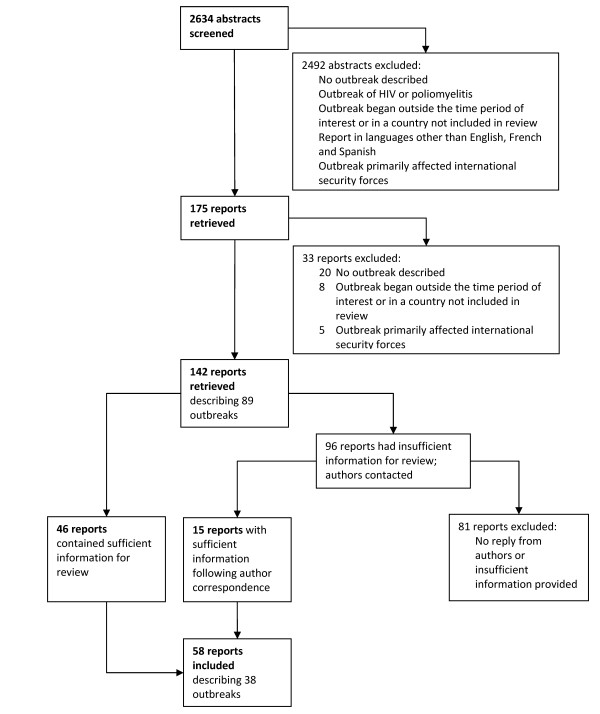
**Search strategy flowchart**.

Of the 2634 abstracts produced by the search strategy, 58 reports describing 38 separate outbreaks were found eligible (Figure 1), of which 35 contained information about mode of detection and 24 about time from onset to detection and/or from detection to further events. Eleven outbreaks occurred in Sudan (including Southern Sudan), four each in the Democratic Republic of the Congo (DRC) and Guinea, three each in Afghanistan, Chad, Myanmar and the Republic of the Congo, and one each in Burundi, Liberia, the Central African Republic, Haiti, Somalia, Angola and Zimbabwe. The aetiologic agents included *Vibrio cholerae *(2), *Plasmodium falciparum *malaria (3), *Neisseria meningitidis *(2), measles virus (3), hepatitis E virus (2), *Shigella dysenteriae *type 1 (1), *Leishmania donovani *(1), yellow fever virus (7), dengue virus (1), Ebola virus (3), unspecified viral haemorrhagic fever (1), scurvy (1), Marburg virus (1), *Escherichia coli *(1), influenza virus (1), *Yersinia pestis *(1), *Tunga penetrans *(1), *Gnathostoma spinigerum *(1), monkeypox virus (1), Rift Valley fever virus (1), West Nile virus, *Salmonella typhi *(1), and *Borrelia *spp. (1).

Of the 58 reports included in the review, 20 were primarily authored by the World Health Organization; 14 by Médecins Sans Frontières; six by journalists; five by international research institutes; three by national research institutes; three by the United States Centers for Disease Control and Prevention; two by overseas governments; two by other NGOs; two by UNICEF; and one by the national government.

### Mode of detection of outbreaks

Among the 35 outbreaks for which mode of detection information was available, 20 (57.1%) were detected through existing surveillance systems, with 10 detected by data analysis (Table 2) and 10 by formal notification (Table 3). Fifteen outbreaks (42.9%) were initially detected through informal notifications (Table 4). For three further outbreaks (yellow fever virus in Guinea, Bounouma subprefecture, August 2008 [16]; *Salmonella typhi *in central Myanmar, September 2000 [17]; *Borrelia *spp. relapsing fever in Southern Sudan, 2000 [18]), the mode of detection was unclear, but time to detection was available: these are included in the timeliness findings (see below).

**Table 2:** Details on outbreaks detected through data analysis (n = 10)

ID	Country, area, date of onset (references)	Aetiologic agent	Onset to detection (days)	Comments
1	Afghanistan, Kabul, May 2005 [19]	*Vibrio cholerae*		Increased case numbers reported through sentinel surveillance system. A low mortality was attributed to the rapid activation of the surveillance system and a rapid response.

2	Burundi, Kayanza Province, Sep 2000 [20-22]	*Plasmodium falciparum*	(11)	Médecins Sans Frontières (MSF) initially noticed a doubling of caseloads over the previous week and compared incidence to previous 3 years. The outbreak was not confirmed until seroprevalence tests were performed in week 7 of the epidemic.

3	Chad, Logone Occidental Province, Feb 2000 [23,24]	*Neisseria meningitidis*		Annual peaks of meningococcal meningitis are noted in this region.

4	Chad, Koumra district, Jan 2001 [24]	*Neisseria meningitidis*		No further details were available.

5	DRC, Kinshasa, Jan 2002 [25]	Measles virus		The outbreak was detected by a sentinel surveillance system. Detection was through both trend analysis and reports from health facilities not included in the system. During the outbreak there were significant delays in reporting from health districts. Limited population movement within the city delayed spread of the epidemic. Early reactive vaccination of unaffected districts could have averted many cases.

6	Sudan, Mornay village and camp, West Darfur, Jul 2004 [26-28]	Hepatatis E virus		The population of Mornay had recently increased due to the arrival of tens of thousands of internally displaced persons. Security concerns and a lack of confidence in Western medicine may have delayed detection. The local hospital became overwhelmed. Cases were reported to the EWARN system.

7	Sudan, northern Sudan, Oct 2003 [29]	Measles virus		Detection was extremely late, almost once the outbreak was over. The investigation pointed to ongoing underreporting of measles by existing surveillance systems in Sudan. Poor access to health-care facilities may be a strong contributing factor.

8	Sudan, Aweil East county (Southern Sudan), Jun 2003 [20]	*Plasmodium falciparum*	(7)	MSF reported an alert after quadrupling of cases. Historical comparisons were hampered by changes in diagnostic strategies and reduced health care utilisation rates due to flooding. Weekly reporting and analysis, and a free and steady supply of anti-malarials may have favoured early detection.

9	Sudan, Abou Shouk camp, North Darfur, Jun 2004 [30]	*Shigella dysenteriae type 1*	46	In the early stages of camp administration, there was poor reporting of diseases. An emergency meeting was held to discuss the number of diarrhoea cases being seen in therapeutic feeding centres and at camp clinics. The WHO's EWARN system verified the outbreak.

10	Sudan, Southern Sudan, Sep 2002 [31-33]	*Leishmania donovani*		Recently internally displaced populations had poor access to health care. Cases were carried on stretchers for days to receive treatment.

* Investigation revealed previously undetected or undiagnosed outbreaks; () indicates that dates were estimated.

**Table 3:** Details on outbreaks detected through formal notifications (n = 10)

ID	Country, area, date of onset (references)	Aetiologic agent	Onset to detection (days)	Comments
11	Guinea, Dinguiraye prefecture, Oct 2004 [34]	Yellow fever virus		In 2002 an African network of laboratories for the diagnosis of yellow fever was developed, leading to far greater testing of acute jaundice cases.

12	Guinea, Kissidougou district, Jun 2006 [35]	Yellow fever virus		A yellow fever vaccination campaign had been conducted in this district, with reported coverage of 93%. Only one case of yellow fever was identified. Close surveillance was to be maintained but a mass vaccination campaign was not considered necessary.

13	Guinea, Faranah health district, Dec 2008 [36]	Yellow fever virus	60	Two cases of yellow fever were initially reported through the yellow fever surveillance system. A further 21 suspected cases were recorded. A targeted mass reactive vaccination campaign was planned.

14	Liberia, Feb 2004 [34]	Yellow fever virus		42 cases of yellow fever were notified from eight of the country's fifteen counties.

15	Myanmar, Yangon, 2001 [37]	Dengue virus		Dengue is endemic in Myanmar. Outbreaks occur cyclically but this outbreak was the largest on record.

16	Republic of Congo, Mbomo and Kelle, Jan 2003 [38-40]	Ebola virus	(34)	In early January 2003, a WHO team arrived in the area to reactivate surveillance and reinforce hygiene promotion, following detection of a zootic among primates. A human outbreak was notified to the Ministry of Health and WHO 15 days later, 7 days after the index case was admitted to hospital. Control efforts were hampered by difficulties in communication and transport. Difficulties with community acceptance were also reported, including strong cultural objections to the collection of blood and post-mortem skin samples, delaying outbreak confirmation.

17	Sudan, Southern Sudan, 2000 [18]	Viral haemorrhagic fever	(7)	A local team from the southern Sudan EWARN detected and reported the case. Test results were available within 2 weeks of the reported onset.

18	Sudan, Torit County (Southern Sudan), May 2003 [41,42]	Yellow fever virus		A Norwegian NGO reported the suspected outbreak through the Southern Sudan EWARN system.

19	Sudan, South Kordofan state, Oct 2005 [43]	Yellow fever virus	(30)	A sentinel surveillance system of hospitals and clinics was in place. Jaundice cases were reported promptly by state health officers through the central surveillance system, but yellow fever was not initially considered and the outbreak was initially attributed to dengue. Laboratory investigation was not initially pursued. Confirmation and the start of control occurred more than a month after notification.

20	Sudan, Yambio county, Southern Sudan, May 2004 [44-47]	Ebola virus	21	Surveillance using haemorrhagic fever case definitions and a rapid response through EWARN contributed to a small number of cases. A concomitant measles outbreak complicated case identification, hampering control measures. On site laboratory facilities could have prevented this.

* Investigation revealed previously undetected or undiagnosed outbreaks; () indicates that dates were estimated.

**Table 4:** Details on outbreaks detected through informal notifications (n = 15)

ID	Country, area, date of onset (references)	Aetiologic agent	Onset to detection (days)	Comments
21	Afghanistan, Bamian, Sep 2000 [48]	*Plasmodium falciparum*	*	A United Nations radio operator notified the alert. A similar outbreak had occurred undetected two to three years earlier.

22	Afghanistan, Taiwara District, Mar 2002 [49]	Scurvy	(46)	Isolation of the district during the winter months delayed detection. The outbreak was reported by an international NGO.

23	Angola, Uige Province, Mar 2005 [50-58]	Marburg virus	*	Concerns of an unusual severe illness were raised by hospital staff in October 2004. A poliomyelitis surveillance officer carried out the initial case investigation in November. Blood samples were sent for analysis at the CDC. Results were initially negative for any viral haemorrhagic fever. Low numbers of similar cases occurred over subsequent months. By 9 March 2005 the situation worsened, and the first death among health care staff occurred. New blood sampling confirmed Marburg on 21 March 2005. Travel by road was precarious, necessitating air transport. Retrospective analysis identified 102 cases dating back to October 2003. Fear and poor adherence to infection control procedures hampered control.

24	Central African Republic, Bangui, Jul 2002 [59]	Hepatitis E virus		A government chief medical officer reported people with jaundice dying of haemorrhage. Yellow fever was initially suspected. Investigation revealed symptoms suggestive of hepatitis. Laboratory tests confirmed Hepatitis E.

25	Chad, Jun 2005 [60]	Measles virus		A senior vaccination officer with MSF noticed a high incidence of measles being reported from health clinics, during a site visit. A surveillance system was in place, but the data were not being analysed.

26	DRC, Kinshasa, Jun 2003 [61]	*Escherichia coli*		An informal alert was raised by the Institut National de Recherche Biomedicale in Kinshasa in response to an increasing incidence of severe diarrhoea testing positive for *E coli*. An outbreak investigation could not be conducted at the time due to political unrest. A high case-fatality amongst infants at a city hospital was attributed to insufficient treatment, particularly haemodialysis, at the beginning of the outbreak.

27	DRC, Bosobolo district, Equateur Province, Nov 2002 [62]	Influenza virus	80	A local NGO reported the outbreak. The area was under the control of a rebel group. The public had little access to medical facilities. A large proportion of deaths could have been prevented with antibiotics.

28	DRC, Orientale Province, Jan 2005 [63]	*Yersinia pestis*	(28)	An informal alert of an epidemic, initially thought to be of haemorrhagic fever, was notified by local health providers in a camp for diamond miners.

29	Haiti, Petites Montagnes, 2004 [64]	*Tunga penetrans*		Health care facilities were up to 20 hours' walk away and at times unreachable. Clinical staff became aware of the outbreak relatively late, after receiving news brought by community health workers.

30	Myanmar, Yangon, 2001 [65]	*Gnathostoma spinigerum*		The outbreak occurred amongst Korean immigrants. The alert was raised by the Korean Embassy.

31	Republic of Congo, Mbomo, Nov 2003 [66,67]	Ebola virus	24	Red Cross volunteers informed local health authorities of a rumour of four suspicious deaths. A week later, a regional investigation team notified an alert of viral haemorrhagic fever to the central level. Impassable roads delayed the response team's arrival by 4 days. The response team was blamed for people dying and for bringing the disease. There was fear of isolation centres and at-home isolation kits were experimented with.

32	Republic of Congo, Impfondo, Likouala district, Jun 2003 [68]	Monkeypox virus	(65)	A physician treated several patients with pox-like lesions over a period of 3 weeks. Alarmed by the severity of the more recent cases, he sent photographs to colleagues from a city hospital of whom one was invited to assist with diagnosis and control. A week later, the outbreak was reported to the CDC and US embassy.

33	Somalia, Afmadow district, Lower Juba Region, Dec 2006 [69]	Rift Valley fever virus		In November 2006, warnings were issued of possible Rift Valley Fever outbreaks, following predictions by spatial models. On 19 December, the WHO received reports of suspected cases in Somalia. Violence, and later also a Kenyan border closure substantially delayed investigation. The virus was laboratory confirmed on 20 January. WHO's outbreak response teams in Nairobi worked closely with poliomyelitis surveillance officers and MSF in Somalia to investigate. Somali medical officers were provided with training on diagnosis and control by the WHO. Security deteriorations further hampered control efforts.

34	Sudan, Nuba mountains, South Kordofan state, 2002 [70]	West Nile virus		MSF operated the only health clinic available in the area, and notified the alert. Cases came from villages up to 8 hours' walk away.

35	Zimbabwe, Aug 2008 [71-73]	*Vibrio cholerae*		Due to collapsing health services, surveillance system completeness was estimated at 30%. The initial recognition of the epidemic was an increased number of cases of 'watery diarrhoea' being noted by Municipal Health Clinics. The ability of the Public Health Laboratory to confirm cholera was greatly limited by shortages of manpower and resources resulting from economic crisis. A second wave of the epidemic from Oct 2008 spread to all provinces and neighbouring countries. The Zimbabwean government declared an epidemic in Dec 2008.

* Investigation revealed previously undetected or undiagnosed outbreaks; () indicates that dates were estimated.

Reports suggested that data analysis proved successful when there was frequent reporting and analysis of data, and with the provision of a free and dependable supply of medication (outbreaks 2, 8). Poor reporting practices delayed detection (outbreak 9). In two instances failures were compensated for by informal notifications after substantial delays (outbreaks 25, 35).

For both data analysis and formal notifications, limited access to and distrust of health services delayed detection (outbreaks 6, 7, 8, 19). In two instances, warnings provided by a geographic information system and detection of an outbreak amongst local wildlife led to enhanced surveillance and eventually detection (outbreaks 16, 33).

Informal notifications originated from a local non-governmental organisation (NGO) (outbreak 27), a research institute (outbreak 26), an embassy (outbreak 30), a UN radio operator (outbreak 21), a rumour received by the WHO (outbreak 33), international NGOs (outbreaks 22, 25, 31, 34), national hospital staff (outbreaks 23, 24, 28, 32). Again, treatment charges and limited access to health facilities delayed detection (outbreaks 25, 27). Four outbreaks occurring within isolated rural communities were associated with late detections (outbreaks 21, 22, 29, 34).

### Timeliness of detection and other events

**Figure 2:**
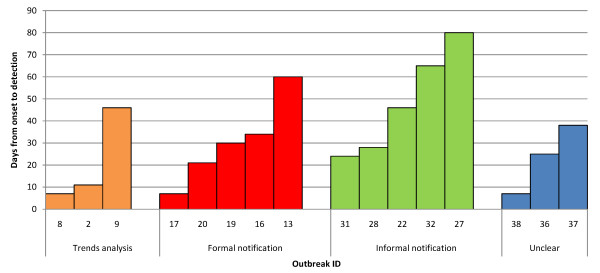
**Delay in days from onset to detection in 15 outbreaks, by mode of detection**.

Overall, the median lag time from onset to detection was 29 days (range 7-80) in 16 outbreaks for which this information was available. In two cases, investigation also unveiled previously undetected and undiagnosed outbreaks due to the same agent. Outbreaks detected through informal notifications appeared to feature the longest detection delays (Figure 2).

**Figure 3:**
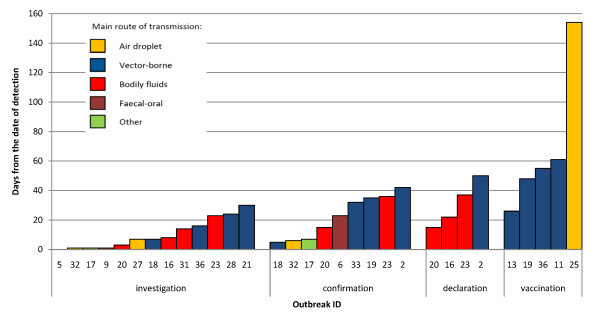
**Delay in days from detection to other events in the outbreak timeline, by main route of transmission of the aetiologic agent**.

From the date of detection, further median (range) delays were 7 days (0-30) to investigation, 23 (5-42) to confirmation, 30 (15-50) to declaration and 55 (26-154) to start of control (reactive vaccination). Numbers were small and no obvious pattern emerged according to the aetiologic agent's route of transmission (Figure 3), but long delays were obvious for some vector-borne disease outbreaks.

Considering the time since reported onset of the outbreak, delays were longer: 42 days (8-87) to investigation in nine outbreaks for which both time to detection and time from detection to investigation were available; 53 (14-71, five outbreaks) to confirmation; 56 (36-61, three outbreaks) to declaration; and 80 (78-86, three outbreaks) to control.

Early warning alert and response network (EWARN) systems set up in southern Sudan and Darfur in 2000 and 2004 respectively, were involved in six Sudanese outbreaks. These outbreaks generally featured the shortest times from onset through to confirmation (outbreaks 6, 9, 17, 18, 20, 38).

Cooperation by communities was greatly hampered by fear and distrust of control teams and biomedical interventions during investigations of Ebola virus and Marburg virus outbreaks (outbreaks 16, 23, 31). Other obstacles to investigation included poor road conditions and insecurity (outbreaks 31, 26, 33). On two occasions, misconceptions by authorities and subsequently late investigations significantly delayed confirmation of causative agents (outbreaks 2, 19).

## Discussion and conclusions

This review suggests that over the last decade surveillance systems have played a considerable role in early outbreak detection in the 22 fragile states included in the review. However, on the whole data analysis seemed to lead to a minority of outbreak detections, with both formal and informal notifications of alerts playing a more prominent, though less timely role. Certain elements of the system played an important role in sensitivity and timeliness, including reduced barriers to health facility utilisation and frequent data analysis. Combining knowledge of the seasonal outbreak risks particular to each area with predictive tools such as geographic information systems could be used to improve the effectiveness of such systems. More importantly, surveillance systems in fragile states should enhance the detection of alerts outside routine data analysis, by focussing more efforts on building both formal and informal networks of informants, particularly where acute emergency conditions or remoteness prevent sophisticated data collection and analysis.

Our review suggested that timeliness of detection, investigation and response is poor for most outbreaks occurring in fragile states, with up to five months elapsing until the start of meaningful control. These delays negate most of the advantages of surveillance and make containment extremely difficult.

Our review is limited by our search strategy, which did not capture outbreaks described in the grey literature. Furthermore, findings may not apply to other states that met fragility criteria for only some of the years within the review's timeframe. Publication bias is likely to influence our findings, but its direction is difficult to gauge: while large outbreaks that were intensively investigated and controlled are more likely to be the subject of publications, small outbreaks that were detected early and contained are probably under-reported. We noted that the vast majority of reports included were authored by institutions based outside the affected countries, with only one report coming from the national ministry of health. This suggests a need to strengthen capacity by fragile states to communicate outbreak surveillance findings, so as to promote ownership of surveillance and outbreak control, and raise the profile of outbreaks and epidemic-prone diseases that international counterparts would not otherwise respond to.

During data abstraction, the considerable heterogeneity of formats and variables included in outbreak reports was apparent. We recommend that a more standardised format be introduced for papers reporting outbreaks, particularly affecting vulnerable populations; and that key meta-data such as the dates of salient events in the outbreak timeline and the circumstances of detection always be reported, so as to enable ongoing global monitoring of the effectiveness of surveillance systems and outbreak control interventions.

## List of abbreviations

DRC: Democratic Republic of Congo; EWARN: Early Warning Alert and Response Network; WHO: World Health Organization; MSF: Médecins Sans Frontières; NGO: Non-governmental organization.

## Competing interests

The authors declare that they have no competing interests.

## Authors' contributions

CB designed the search strategy, carried out the review and co-wrote the paper. FC designed the search strategy and co-wrote the paper. All authors read and approved the final manuscript.

## Supplementary Material

Additional file 1 **Appendix containing details on the selection of countries included in the review and on the search strategy used**. Click here for file
