# Supplementary material for: Detection of infectious disease outbreaks in twenty-two fragile states, 2000-2010: a systematic review
Source: Confl Health. 2011 Aug 23;5:13. doi: 10.1186/1752-1505-5-13 (PMC3180250; doi:10.1186/1752-1505-5-13)
Supplement: Additional file 1 — Appendix containing details on the selection of countries included in the review and on the search strategy used. [file 1752-1505-5-13-S1.DOC]

**Detection of infectious disease outbreaks in twenty-two fragile states, 2000-2010: A systematic review**

Catherine Bruckner, Francesco Checchi

**Additional File 1**

**Selection of fragile states**

In Table S1, shading of country boxes indicates inclusion of country in the review. Shading of boxes in year columns indicates that the fragile states definition was met for that year. Timor Leste was included as it has been an independent country since 2002 only.

Lists of fragile states lists, or low-income countries under stress (LICUS, as fragile states were previously known) as produced by the World Bank were available through the internet for years 2003, 2004, 2005, 2006 and 2007, while an estimated list was provided for 2010. For the years 2008 and 2009, fragile states lists were estimated using available CPIA lists for IDA-eligible countries. The average overall CPIA rating was calculated, and those countries with scores of 3.2 or less were included in the list for that year. CPIA quintile lists for IDA-eligible countries in 2000, 2001 and 2002 were obtained through correspondence with the World Bank. All countries falling within the fourth and fifth quintiles, corresponding to a CPIA score of 3 and below, were considered fragile.

Table S1.

| **Country** | **Year** | | | | | | | | | | |
| --- | --- | --- | --- | --- | --- | --- | --- | --- | --- | --- | --- |
|  | **2000** | **2001** | **2002** | **2003** | **2004** | **2005** | **2006** | **2007** | **2008** | **2009** | **2010** |
| Afghanistan |  |  |  |  |  |  |  |  |  |  |  |
| Albania |  |  |  |  |  |  |  |  |  |  |  |
| Angola |  |  |  |  |  |  |  |  |  |  |  |
| Armenia |  |  |  |  |  |  |  |  |  |  |  |
| Azerbaijan |  |  |  |  |  |  |  |  |  |  |  |
| Bangladesh |  |  |  |  |  |  |  |  |  |  |  |
| Benin |  |  |  |  |  |  |  |  |  |  |  |
| Bhutan |  |  |  |  |  |  |  |  |  |  |  |
| Bolivia |  |  |  |  |  |  |  |  |  |  |  |
| Bosnia and Herzegovina |  |  |  |  |  |  |  |  |  |  |  |
| Burkina Faso |  |  |  |  |  |  |  |  |  |  |  |
| Burundi |  |  |  |  |  |  |  |  |  |  |  |
| Cambodia |  |  |  |  |  |  |  |  |  |  |  |
| Cameroon |  |  |  |  |  |  |  |  |  |  |  |
| Cape Verde |  |  |  |  |  |  |  |  |  |  |  |
| Central African Republic |  |  |  |  |  |  |  |  |  |  |  |
| Chad |  |  |  |  |  |  |  |  |  |  |  |
| Comoros |  |  |  |  |  |  |  |  |  |  |  |
| Congo, Dem. Rep. of |  |  |  |  |  |  |  |  |  |  |  |
| Congo, Rep. |  |  |  |  |  |  |  |  |  |  |  |
| Cote d'Ivoire |  |  |  |  |  |  |  |  |  |  |  |
| Djibouti |  |  |  |  |  |  |  |  |  |  |  |
| Dominica |  |  |  |  |  |  |  |  |  |  |  |
| Equatorial Guinea |  |  |  |  |  |  |  |  |  |  |  |
| Eritrea |  |  |  |  |  |  |  |  |  |  |  |
| Ethiopia |  |  |  |  |  |  |  |  |  |  |  |
| Gambia, The |  |  |  |  |  |  |  |  |  |  |  |
| Georgia |  |  |  |  |  |  |  |  |  |  |  |
| Ghana |  |  |  |  |  |  |  |  |  |  |  |
| Grenada |  |  |  |  |  |  |  |  |  |  |  |
| Guinea |  |  |  |  |  |  |  |  |  |  |  |
| Guinea-Bissau |  |  |  |  |  |  |  |  |  |  |  |
| Guyana |  |  |  |  |  |  |  |  |  |  |  |
| Haiti |  |  |  |  |  |  |  |  |  |  |  |
| Honduras |  |  |  |  |  |  |  |  |  |  |  |
| India |  |  |  |  |  |  |  |  |  |  |  |
| Indonesia |  |  |  |  |  |  |  |  |  |  |  |
| Kenya |  |  |  |  |  |  |  |  |  |  |  |
| Kiribati |  |  |  |  |  |  |  |  |  |  |  |
| Kosovo |  |  |  |  |  |  |  |  |  |  |  |
| Kyrgyz Republic |  |  |  |  |  |  |  |  |  |  |  |
| Lao P.D.R. |  |  |  |  |  |  |  |  |  |  |  |
| Lesotho |  |  |  |  |  |  |  |  |  |  |  |
| Liberia |  |  |  |  |  |  |  |  |  |  |  |
| Madagascar |  |  |  |  |  |  |  |  |  |  |  |
| Malawi |  |  |  |  |  |  |  |  |  |  |  |
| Maldives |  |  |  |  |  |  |  |  |  |  |  |
| Mali |  |  |  |  |  |  |  |  |  |  |  |
| Mauritania |  |  |  |  |  |  |  |  |  |  |  |
| Moldova |  |  |  |  |  |  |  |  |  |  |  |
| Mongolia |  |  |  |  |  |  |  |  |  |  |  |
| Mozambique |  |  |  |  |  |  |  |  |  |  |  |
| Myanmar |  |  |  |  |  |  |  |  |  |  |  |
| Nepal |  |  |  |  |  |  |  |  |  |  |  |
| Nicaragua |  |  |  |  |  |  |  |  |  |  |  |
| Niger |  |  |  |  |  |  |  |  |  |  |  |
| Nigeria |  |  |  |  |  |  |  |  |  |  |  |
| Pakistan |  |  |  |  |  |  |  |  |  |  |  |
| Papua New Guinea |  |  |  |  |  |  |  |  |  |  |  |
| Rwanda |  |  |  |  |  |  |  |  |  |  |  |
| Samoa |  |  |  |  |  |  |  |  |  |  |  |
| Sao Tome and Principe |  |  |  |  |  |  |  |  |  |  |  |
| Senegal |  |  |  |  |  |  |  |  |  |  |  |
| Serbia |  |  |  |  |  |  |  |  |  |  |  |
| Sierra Leone |  |  |  |  |  |  |  |  |  |  |  |
| Solomon Islands |  |  |  |  |  |  |  |  |  |  |  |
| Somalia |  |  |  |  |  |  |  |  |  |  |  |
| Sri Lanka |  |  |  |  |  |  |  |  |  |  |  |
| St. Lucia |  |  |  |  |  |  |  |  |  |  |  |
| St. Vincent and the Grenadines |  |  |  |  |  |  |  |  |  |  |  |
| Sudan |  |  |  |  |  |  |  |  |  |  |  |
| Tajikistan |  |  |  |  |  |  |  |  |  |  |  |
| Tanzania |  |  |  |  |  |  |  |  |  |  |  |
| Timor-Leste |  |  |  |  |  |  |  |  |  |  |  |
| Togo |  |  |  |  |  |  |  |  |  |  |  |
| Tonga |  |  |  |  |  |  |  |  |  |  |  |
| Uganda |  |  |  |  |  |  |  |  |  |  |  |
| Uzbekistan |  |  |  |  |  |  |  |  |  |  |  |
| Vanuatu |  |  |  |  |  |  |  |  |  |  |  |
| Vietnam |  |  |  |  |  |  |  |  |  |  |  |
| Yemen, Rep. of |  |  |  |  |  |  |  |  |  |  |  |
| Zambia |  |  |  |  |  |  |  |  |  |  |  |
| Zimbabwe |  |  |  |  |  |  |  |  |  |  |  |

**OvidSP search strategy**

1. (Bacillary dysentery or shigellosis or dysenterie bacillaire or shigellose or dysenterie or disenteria bacilar or shigelosis or disenteria).mp. [mp=ti, ab, sh, hw, tn, ot, dm, mf, bt, nm, ui]

2. limit 1 to (humans and yr="2000 -Current")

3. (cholera or Colera).mp. [mp=ti, ab, sh, hw, tn, ot, dm, mf, bt, nm, ui]

4. limit 3 to (humans and yr="2000 -Current")

5. (Hepatitis or acute hepatitis or Hepatite or hepatite aigue).mp. [mp=ti, ab, sh, hw, tn, ot, dm, mf, bt, nm, ui]

6. limit 5 to (humans and yr="2000 -Current")

7. (Malaria or Paludisme).mp. [mp=ti, ab, sh, hw, tn, ot, dm, mf, bt, nm, ui]

8. limit 7 to (humans and yr="2000 -Current")

9. (measles or Rougeole or Sarampion).mp. [mp=ti, ab, sh, hw, tn, ot, dm, mf, bt, nm, ui]

10. limit 9 to (humans and yr="2000 -Current")

11. (Meningococcal disease or meningitis or meningite or maladie a meningocoques or la meningitis or enfermedad meningococica).mp. [mp=ti, ab, sh, hw, tn, ot, dm, mf, bt, nm, ui]

12. limit 11 to (humans and yr="2000 -Current")

13. (Typhoid fever or typhoide or tifoidea).mp. [mp=ti, ab, sh, hw, tn, ot, dm, mf, bt, nm, ui]

14. limit 13 to (humans and yr="2000 -Current")

15. (viral haemorrhagic fever or Ebola or Lassa fever or Marburg or fievre hemorragique virale or fievre de Lassa or Fiebre hemorragica viral or fiebre de Lassa or ebola).mp. [mp=ti, ab, sh, hw, tn, ot, dm, mf, bt, nm, ui]

16. limit 15 to (humans and yr="2000 -Current")

17. (yellow fever or Fievre jaune or Fiebre amarilla).mp. [mp=ti, ab, sh, hw, tn, ot, dm, mf, bt, nm, ui]

18. limit 17 to (humans and yr="2000 -Current")

19. (Dengue fever or Dengue Haemorrhagic fever or dengue or dengue hemorragique).mp. [mp=ti, ab, sh, hw, tn, ot, dm, mf, bt, nm, ui]

20. limit 19 to (humans and yr="2000 -Current")

21. (Rift Valley fever or Fievre de la Vallee du Rift or Fiebre del Valle del Rift).mp. [mp=ti, ab, sh, hw, tn, ot, dm, mf, bt, nm, ui]

22. limit 21 to (humans and yr="2000 -Current")

23. (Polio or Poliomyelitis or poliomyelite).mp. [mp=ti, ab, sh, hw, tn, ot, dm, mf, bt, nm, ui]

24. limit 23 to (humans and yr="2000 -Current")

25. (Avian Flu or Avian influenza or Grippe aviaire or Gripe aviar).mp. [mp=ti, ab, sh, hw, tn, ot, dm, mf, bt, nm, ui]

26. limit 25 to (humans and yr="2000 -Current")

27. (outbreak or declenchement or brote).mp. [mp=ti, ab, sh, hw, tn, ot, dm, mf, bt, nm, ui]

28. limit 27 to (humans and yr="2000 -Current")

29. (epidemic or epidemie or epidemia).mp. [mp=ti, ab, sh, hw, tn, ot, dm, mf, bt, nm, ui]

30. limit 29 to (humans and yr="2000 -Current")

31. 2 or 4 or 6 or 8 or 10 or 12 or 14 or 16 or 18 or 20 or 22 or 24 or 26 or 28 or 30

32. (outbreak or detection or cases or epidemic or apparition or detection or epidemie or cas or foco or deteccion or casos or epidemia).mp. [mp=ti, ab, sh, hw, tn, ot, dm, mf, bt, nm, ui]

33. limit 32 to (humans and yr="2000 -Current")

34. 31 and 33

35. (Afghanistan or Afganistan).mp. [mp=ti, ab, sh, hw, tn, ot, dm, mf, bt, nm, ui]

36. limit 35 to human

37. limit 36 to yr="2000 -Current"

38. limit 37 to humans

39. 34 and 38

40. remove duplicates from 39

41. Angola.mp. [mp=ti, ab, sh, hw, tn, ot, dm, mf, bt, nm, ui]

42. limit 41 to human

43. limit 42 to yr="2000 -Current"

44. limit 43 to humans

45. 34 and 44

46. remove duplicates from 45

47. Burundi.mp. [mp=ti, ab, sh, hw, tn, ot, dm, mf, bt, nm, ui]

48. limit 47 to human

49. limit 48 to yr="2000 -Current"

50. limit 49 to humans

51. 34 and 50

52. remove duplicates from 51

53. (Central African Republic or Republique Centrafricaine or Republica Central Africana).mp. [mp=ti, ab, sh, hw, tn, ot, dm, mf, bt, nm, ui]

54. limit 53 to human

55. limit 54 to yr="2000 -Current"

56. limit 55 to humans

57. 34 and 56

58. remove duplicates from 57

59. (Chad or Tchad).mp. [mp=ti, ab, sh, hw, tn, ot, dm, mf, bt, nm, ui]

60. limit 59 to human

61. limit 60 to yr="2000 -Current"

62. limit 61 to humans

63. 34 and 62

64. remove duplicates from 63

65. (Comoros or Comores or Comoras).mp. [mp=ti, ab, sh, hw, tn, ot, dm, mf, bt, nm, ui]

66. limit 65 to human

67. limit 66 to yr="2000 -Current"

68. limit 67 to humans

69. 34 and 68

70. remove duplicates from 69

71. (Democratic Republic of Congo or DRC or Zaire or Republique democratique du Congo or Republica Democratica del Congo).mp. [mp=ti, ab, sh, hw, tn, ot, dm, mf, bt, nm, ui]

72. limit 71 to human

73. limit 72 to yr="2000 -Current"

74. limit 73 to humans

75. 34 and 74

76. remove duplicates from 75

77. (Republic of Congo or Republique du Congo or Republica del Congo).mp. [mp=ti, ab, sh, hw, tn, ot, dm, mf, bt, nm, ui]

78. limit 77 to human

79. limit 78 to yr="2000 -Current"

80. limit 79 to humans

81. 34 and 80

82. remove duplicates from 81

83. (Guinea or Guinee).mp. [mp=ti, ab, sh, hw, tn, ot, dm, mf, bt, nm, ui]

84. limit 83 to human

85. limit 84 to yr="2004 -Current"

86. limit 85 to humans

87. 34 and 86

88. remove duplicates from 87

89. (Guinea-Bissau or Guinee-Bissau).mp. [mp=ti, ab, sh, hw, tn, ot, dm, mf, bt, nm, ui]

90. limit 89 to human

91. limit 90 to yr="2000 -Current"

92. limit 91 to humans

93. 34 and 92

94. remove duplicates from 93

95. Haiti.mp. [mp=ti, ab, sh, hw, tn, ot, dm, mf, bt, nm, ui]

96. limit 95 to human

97. limit 96 to yr="2000 -Current"

98. limit 97 to humans

99. 34 and 98

100. remove duplicates from 99

101. 31 and 33

102. Liberia.mp. [mp=ti, ab, sh, hw, tn, ot, dm, mf, bt, nm, ui]

103. limit 102 to human

104. limit 103 to yr="2003 -Current"

105. limit 104 to humans

106. 101 and 105

107. remove duplicates from 106

108. (Myanmar or Burma or Birmanie or Birmania).mp. [mp=ti, ab, sh, hw, tn, ot, dm, mf, bt, nm, ui]

109. limit 108 to human

110. limit 109 to yr="2003 -Current"

111. limit 110 to humans

112. 101 and 111

113. remove duplicates from 112

114. (Sao Tome Principe or Sao Tome-et-Principe or Santo Tome y Principe).mp. [mp=ti, ab, sh, hw, tn, ot, dm, mf, bt, nm, ui]

115. limit 114 to human

116. limit 115 to yr="2004 -Current"

117. limit 116 to humans

118. 101 and 117

119. Sierra Leone.mp. [mp=ti, ab, sh, hw, tn, ot, dm, mf, bt, nm, ui]

120. limit 119 to human

121. limit 120 to yr="2000 -Current"

122. limit 121 to humans

123. 101 and 122

124. remove duplicates from 123

125. (Solomon Islands or Iles Salomon or Islas Salomon).mp. [mp=ti, ab, sh, hw, tn, ot, dm, mf, bt, nm, ui]

126. limit 125 to human

127. limit 126 to yr="2004 -Current"

128. limit 127 to humans

129. 101 and 128

130. remove duplicates from 129

131. (Somalia or Somalie).mp. [mp=ti, ab, sh, hw, tn, ot, dm, mf, bt, nm, ui]

132. limit 131 to human

133. limit 132 to yr="2000 -Current"

134. limit 133 to humans

135. 101 and 134

136. remove duplicates from 135

137. (Sudan or Soudan).mp. [mp=ti, ab, sh, hw, tn, ot, dm, mf, bt, nm, ui]

138. limit 137 to human

139. limit 138 to yr="2000 -Current"

140. limit 139 to humans

141. 101 and 140

142. remove duplicates from 141

143. (Tajikistan or Tadjikistan or Tayikistan).mp. [mp=ti, ab, sh, hw, tn, ot, dm, mf, bt, nm, ui]

144. limit 143 to human

145. limit 144 to yr="2003 -Current"

146. limit 145 to humans

147. 101 and 146

148. remove duplicates from 147

149. (Timor-Leste or East Timor).mp. [mp=ti, ab, sh, hw, tn, ot, dm, mf, bt, nm, ui]

150. limit 149 to human

151. limit 150 to yr="2004 -Current"

152. limit 151 to humans

153. 101 and 152

154. remove duplicates from 153

155. Togo.mp. [mp=ti, ab, sh, hw, tn, ot, dm, mf, bt, nm, ui]

156. limit 155 to human

157. limit 156 to yr="2000 -Current"

158. limit 157 to humans

159. 101 and 158

160. remove duplicates from 159

161. Zimbabwe.mp. [mp=ti, ab, sh, hw, tn, ot, dm, mf, bt, nm, ui]

162. limit 161 to human

163. limit 162 to yr="2000 -Current"

164. limit 163 to humans

165. 101 and 164

166. remove duplicates from 165
